# Addressing Health Disparities in the Rural United States: Advocacy as Caregiving among Community Health Workers and *Promotores de Salud*

**DOI:** 10.3390/ijerph17249223

**Published:** 2020-12-10

**Authors:** Ryan I. Logan, Heide Castañeda

**Affiliations:** 1 Department of Anthropology, Geography & Ethnic Studies, California State University Stanislaus, Turlock, CA 95382, USA; 2 Department of Anthropology, University of South Florida, Tampa, FL 33620, USA; hcastaneda@usf.edu

**Keywords:** community health workers, *promotores de salud*, rural health, health disparities, advocacy, care, caregiving, COVID-19, pandemic, Indiana, Texas

## Abstract

Rural populations in the United States are faced with a variety of health disparities that complicate access to care. Community health workers (CHWs) and their Spanish-speaking counterparts, *promotores de salud*, are well-equipped to address rural health access issues, provide education, and ultimately assuage these disparities. In this article, we compare community health workers in the states of Indiana and Texas, based on the results of two separate research studies, in order to (1) investigate the unique role of CHWs in rural communities and (2) understand how their advocacy efforts represent a central form of caregiving. Drawing on ethnographic, qualitative data—including interviews, photovoice, and participant observation—we analyze how CHWs connect structurally vulnerable clients in rural areas to resources, health education, and health and social services. Our primary contribution to existing scholarship on CHWs is the elaboration of advocacy as a form of caregiving to improve individual health outcomes as well as provoke structural change in the form of policy development. Finally, we describe how CHWs became especially critical in addressing disparities among rural populations in the wake of COVID-19, using their advocacy-as-caregiving role that was developed and well-established before the pandemic. These frontline workers are more vital than ever to address disparities and are a critical force in overcoming structural vulnerability and inequities in health in the United States.

## 1. Introduction

Understanding how health disparities affect populations living in rural areas—especially as these areas are becoming increasingly diverse [1,2,3]—is essential to the broader promotion of health and well-being. Various structural forces, including poverty, transportation barriers, and a lack of accessible medical facilities and providers [3,4,5], deleteriously impact those living in rural areas, thereby complicating access to care and negatively affecting health outcomes [6]. Additional social determinants of health and structural vulnerabilities impact marginalized populations based on their socioeconomic status and racial/ethnic identification [7].

In spite of these issues, community health workers (CHWs) and their Spanish-speaking counterparts, *promotores/as de salud* (literally, “health promoters”; henceforth, promotores), work to circumvent structural barriers for clients (this was the preferred term by CHWs in Indiana to refer to the individuals to whom they provided services) and communities. CHWs and promotores are central to the health care workforce in many countries throughout the world, as well as in many urban and rural environments throughout the United States [8,9,10,11,12]. They enhance access to care, provide health education, spend the majority of their time within the community (of which they are typically members) instead of in the clinic, and participate in advocacy activities on behalf of their communities. As such, CHWs are a critical force in overcoming structural vulnerability and inequities in health in the United States.

In this article, we examine CHWs in the states of Indiana and Texas to compare and contrast their activities and experiences in each of these rural settings. Our analysis is based on the results of two separate research studies; both projects shared the same ethnographic, qualitative approach and included community members and CHWs as research partners. The questions guiding our analysis were: (1) what is the unique role of CHWs in rural communities, and (2) how do their advocacy efforts represent a central form of caregiving? Advocacy, we argue, is central to their role as champions of the health of clients in addition to positively transforming the broader health landscape through policy efforts at the organizational, state, and national levels. However, this advocacy aspect of their duties within the health system has been left largely understudied, with most research focusing instead on their health education or (linguistic and clinical) translational activities.

We present two cases from our respective research sites, the first situated in rural Indiana and the other located in the Rio Grande Valley (RGV) of southern Texas. While the first site is predominantly White, there is sizable and increasing number of Spanish-speaking migrants; the second is situated in the U.S.–Mexico borderlands with a fairly homogenous Mexican-origin and predominantly rural, Spanish-speaking population. We explore the ways in which these workers encounter similar and distinct issues in each location, how they address gaps in care, and the ways in which CHWs and promotores circumvent health disparities to improve health for marginalized populations and provoke positive health outcomes in these rural settings.

Despite the geographic distance and different local histories in the settings, these two case studies also offer many similarities for analysis. Most notably, these communities are faced with a variety of social determinants of health, including poverty, lack of transportation, a shortage of medical providers, and language barriers, all of which frequently lead to an inability to carry out treatment plans. However, as these cases highlight, CHWs and promotores assuaged many of these issues through direct interaction with clients and by disseminating health education within the community. In particular, our analysis highlights their important role as advocates. In aiding individual clients and drawing attention to local health disparities, these workers actively seek change by working directly with policy makers. We argue that this advocacy role must be considered central to the activities of CHWs and promotores to effectively address gaps in the provision of care in rural communities. Through their unique abilities and approaches, these workers circumvent the impact of structural violence, and empower communities to secure health and well-being. Especially as the COVID-19 pandemic has exacerbated health disparities, these frontline workers are pivotal in addressing structural barriers and health care needs.

## 2. Materials and Methods

Our projects draw on ethnographic, qualitative data collection strategies particularly well-suited for research with CHWs and promotores, since they can elucidate the constellation of factors that impact caregiving, relationships with clients and stakeholders, and areas for policy development [13]. We follow the suggestion of scholars who have called for the inclusion of CHWs and promotores as research partners [13,14,15,16,17], especially as they share a close connection with the communities they serve and can leverage these relationships for data collection when the presence of researchers may otherwise be disruptive. In the Results section below, the names and titles of organizations in this article are pseudonyms.

### 2.1. Data Collection

Both studies documented in this article utilized semi-structured interviews and participant observation, and both collected follow-up data in 2020 related to COVID-19 activities and experiences. Even though this was not part of either project’s original research questions, it was considered as an important component of the longitudinal design and ongoing commitment to understanding emergent phenomena in each respective community.

Logan (Author 1) drew on a collaborative framework with a local CHW organization in Indiana, interviewing a total of 49 CHWs, collecting more than 300 hours of participant observation, in addition to completing a photovoice project that lasted several months. Primary data collection occurred for eleven consecutive months between 2017 and 2018. Participants also took part in initial data analysis in the form of a focus group interview, in which Logan presented findings and elicited feedback and critique. In 2019, he completed follow-up interviews with thirteen participants and two new participants. Following the onset of the COVID-19 pandemic, additional participant observation data were collected in the form of public Zoom meetings held by the partner CHW organization throughout 2020.

Data for the South Texas case study were drawn from a series of projects over a five-year period from 2013 to 2017. These included interviews with 62 health care providers, community health workers/promotores, social workers, non-profit organizational staff, public health officials, and other key stakeholders. These interviews provided information about resources and major challenges and successes in the region, including how they related to the work on CHWs in rural areas of the state. The projects also collected 252 interviews with mixed-status immigrant families in the region, many of whom essentially represented the clients of CHWs. These data were supplemented by extensive on-the-ground participant observation across five years, including spending time in homes and clinical settings as well as attending a variety of community events. Follow-up interviews with seven promotores were conducted in 2018 and in 2020, the latter focusing on responses to COVID-19 in the community.

Both projects received human subject approval from the University of South Florida (IRB Studies #00020583 and #00030835) to conduct this research.

### 2.2. Analysis

Both studies analyzed their respective data using MAXQDA, a data analysis software program. Transcripts and notes were entered into MAXQDA and coded to capture domains of interest relevant to each research question. The coding process utilized both deductively derived codes, as well as inductively derived codes emerging from the data and reflecting the responses of participants. Descriptive coding was further utilized to draw out major words, phrases, and concepts and to compare and contrast data points across interviews and across the two projects. For both projects, preliminary analyses were brought back to the organizational partners for critique and validation through summaries and discussions.

### 2.3. Limitations

Both of these research projects were qualitative in design and relied on referral sampling methods. Thus, no generalizability is implied, although the general patterns we describe in the Results section below will be similar in comparable sites and populations.

## 3. Background

### 3.1. Rural Health Disparities

Living in a rural area has been recognized as a unique health disparity [18], resulting in higher rates of chronic disease, lack of access to mental health care, and higher rates of terminal illnesses, including cancer [3,19]. These disparities are compounded for those who are the most structurally vulnerable in these rural areas, such as racial and ethnic minority and immigrant populations. As a result, many must engage in “band-aid care” [20], including informal transactions in care-seeking, including bartering, rationing, noncompliance, and hoarding. This is a response to restrictive federal and state-level policies that impose “uneven geographies” of health care for individuals in rural areas and can complicate caregiving for frontline providers [20].

As a whole, racial and ethnic minority populations in rural communities are exposed to greater health disparities. For example, Latinx migrant farmworkers suffer from higher rates of health disparities and occupational hazards, with access to care exacerbated by a constellation of factors that are legal, financial, cultural, and geographic in nature [21]. Other issues, such as social isolation, deleteriously affect Latinx immigrants and lead to additional barriers to care and a higher incidence of mental health problems [22]. Moreover, these workers may live in deteriorating housing, which can provoke health issues due to mold, mildew, and other allergens [23]. There are significant disparities between White and Black populations in rural areas, with the latter experiencing higher rates of mortality [24].

The COVID-19 pandemic has brought with it additional challenges to rural populations, as disputes over mask mandates and conceptions of immunity in rural populations and on the part of governments of rural states have left residents more vulnerable to infection with the virus [25]. The circumstances within which Black, immigrant, Native American, and incarcerated people live and work in rural areas have also been largely ignored [25,26]. Compounding these issues has been a surge in rates of COVID-19 throughout the rural U.S. [27], with racial and ethnic minority populations disproportionately affected by the pandemic [28,29]. Addressing disparities in rural health and specific outreach to minority populations is thus crucial to improving access to care and promoting positive health outcomes for all residents.

### 3.2. Role of CHWs and Promotores

CHWs and promotores are essential intermediaries and in many ways first responders to the needs of marginalized rural populations. Previous studies have confirmed the positive health outcomes and cost-effectiveness of these workers within rural communities [3,30,31,32,33,34,35]. CHWs and promotores seamlessly maneuver within their communities, connecting clients to resources, improving health access, providing health education, and circumventing the impacts of structural barriers. For instance, CHWs effectively reduced the impacts of type-2 diabetes and improved health education among a sample of Mexican American adults in the RGV [35]. In response to the COVID-19 pandemic, the need for their skills to connect marginalized communities, distribute information, and promote health behaviors has been recognized [36].

Social determinants of health include socioeconomic status, access to food, and physical and environmental safety. These factors are often constructed via policies and institutions and coalesce to influence care and the ability to achieve well-being. Populations living in rural settings suffer from many of the same social determinants of health as those in urban settings. However, in rural settings, these may become exacerbated through a lack of financial capital, lack of transportation, geographic isolation, high rates of chronic disease, high rates of health risk behaviors, low rates of health insurance, and a lack of providers [3,5,37]. Additional factors, such as a lack of access to technology (e.g., the Internet), further complicate issues [38]. The rural setting combined with various social determinants of health produce a negative synergy that perniciously affects the health of individuals and communities. While rural communities are often fairly homogenous in racial and ethnic composition, many areas have become increasingly diverse, and the various factors affecting different populations have been largely overlooked [6].

Compounding the deleterious impacts of the social determinants of health is the structural vulnerability of particular populations. Structural vulnerability is a theoretical framework which assesses the susceptibility of an individual based on their location in the social hierarchy [39]. This lens elucidates how sociodemographic factors (e.g., race, ethnicity, sexuality, gender, immigration status, etc.) affect individuals differently. For immigrant communities living in rural areas, issues related to legal status differentially impact access to care in addition to the ability to afford treatment. Thus, immigration serves as a social determinant of health, which can negatively affect the well-being of these individuals [7]. However, CHWs and promotores can assuage these issues. Given their trusted position in the community and depth of knowledge of local resources, these workers collaborate with clients to find ways to overcome these issues. The ability of these workers to address the impact of social determinants of health is especially vital for immigrant and marginalized communities [40]. The trusting relationships they foster help CHWs connect with clients and collaboratively develop strategies to address social determinants of health [4,40,41]. For individuals living in rural areas and who are affected by varying degrees of structural vulnerability, these workers are essential in promoting health and advocating for the needs of the broader community.

Advocacy is considered an essential, core role of CHWs that is intimately connected to health and well-being in addition to its potential to provoke policy change [4,9,15,42,43,44]. Several studies have explored the positive impacts of advocacy performed by CHWs and promotores [4,43,44]. Advocacy contributes to the health of rural communities by highlighting structural factors that must be addressed to improve access. Through advocacy, these workers address individual and community health and social service needs as well as engaging with local, state, and federal policy makers in promoting strategies to improve the overall well-being of communities [4,42,44]. As such, we argue that advocacy serves a form of caregiving, improving access, health, and overall well-being through positive health behavior change, engendering empowerment, and policy development. However, this aspect of the work of CHWs and promotores has been largely understudied, as most research focused instead on their health education or (linguistic and clinical) translational activities.

### 3.3. Characteristics of Data Collection Sites

Both Indiana and Texas have significant poor indicators of population health. Indiana ranks as 41st out of 50 in terms of overall health [45]. Additionally, the state ranks poorly in several other health areas, including infant mortality (43rd), premature death (39th), diabetes (36th), frequent mental distress (34th), frequent physical distress (32nd), and preventable hospitalizations (41st) [45]. These factors are compounded by the fact that the state government only spends USD 53 per capita on public health (47th) [45]. Similarly, Texas has significant health issues, ranking 34th in terms of overall health [46]. Primary health issues in the state include low numbers of mental health providers (49th), primary care providers (45th), high rates of preventable hospitalizations (34th), diabetes (40th), and the percentage of the population that is uninsured (50th). Texas only ranks one spot above Indiana in terms of public health spending per capita, at USD 60 (40th) [46]. These statistics highlight not only that the two states are comparable for analysis but also confirms that the health issues in both states are well-suited to be addressed via the work of CHWs and promotores.

Moreover, both Indiana and Texas trend politically conservative and have introduced specific policies that have led to deleterious outcomes for marginalized groups, including Asian, Black, Latinx, and immigrant populations, and individuals experiencing homelessness, mental health disorders, and/or substance abuse disorders. Although Indiana expanded Medicaid in 2014, those enrolled—who already face economic insecurity—are required to pay a portion of their insurance, with failure to do so resulting in disenrollment for up to six months [47]. Lack of progress in addressing the opioid epidemic, failure to provide HIV screenings, and political opposition to needle exchange programs saw the highest rates of new HIV infections in Indiana, reaching its apex in 2015 [48,49]. Furthermore, Indiana participates in the Secure Communities program, in which local law enforcement agencies collaborate with federal immigration agents, creating an oppressive atmosphere for undocumented immigrants [50].

Meanwhile, the debate over affordable health care in Texas has been highly polarized and the state remains in adamant opposition to implementing the Affordable Care Act. Lawmakers rejected the expansion of adult Medicaid, leaving one million low-income working adults caught in the ‘coverage gap’, in which their incomes exceeded eligibility criteria but were too low to qualify them for subsidies to assist in purchasing insurance through the exchanges. Low-income Texas residents with incomes between 100% and 138% of the federal poverty level were provided with a new option to purchase subsidized private coverage through the federally facilitated Marketplace, and federal matching funds were made available to increase outreach. However, political opposition led lawmakers to reduce the availability of application assistance, as the state imposed strict regulations on navigators—a major role for many community health workers and promotores—who work in the community to explain coverage options and enroll people in health plans. As a result, in Texas, only 35% of the potential Marketplace population enrolled, leaving some 2 million eligible people without insurance coverage [51]. A decline in enrollment in the Children’s Health Insurance Program (CHIP) for mixed-status immigrant families occurred with aggressive immigration enforcement under the Trump administration, as well as with the passage of Senate Bill 4, which allows local law enforcement officers to investigate the legal status of those they detain. Furthermore, women’s health care and family planning have been increasingly difficult to access in Texas, especially in rural areas such as the Rio Grande Valley. In 2011, the state legislature slashed the state’s family planning funding for low-income women by two thirds and authorized the “affiliate rule,” which denied federal family planning funds to Planned Parenthood because it provided abortion care. Within 2 years, 28% of family planning clinics in the Rio Grande Valley had closed and many others were forced to cut services [52] (p. 6).

## 4. Results

The following sections detail findings from Indiana and Texas to illustrate the work of CHWs or promotores specifically in alleviating issues for rural populations. These case studies present similar issues encountered, despite different geographic locations, as well as how these medical paraprofessionals have worked to address gaps in care and enhance health and overall well-being. We pay particular attention to advocacy efforts on behalf of individual clients, within the broader community, and with stakeholders and policy makers in order to elucidate how this represents a form of caregiving. We have structured our presentation of findings around three major themes that emerged from data analysis in each study relating to CHWs addressing: (1) social determinants of health specifically related to rural settings; (2) immigration issues; and (3) the COVID-19 pandemic. In each section, we highlight CHW advocacy as a primary tool and draw comparisons in the following Discussion.

### 4.1. CHWs and Social Determinants of Health in Rural Indiana

In Indiana, CHWs provide direct services in diverse communities in rural settings, working with White, Black, Latinx, and Asian populations as well as with individuals experiencing homelessness, mental health disorders, and/or substance abuse disorders. There were several common social determinants of health that negatively impacted access and led to deleterious outcomes for residents living in rural areas. Jane, a CHW who works in rural, north-central Indiana, explained that a major issue affecting her clients was a lack of access to technology. She explained:

A lot of the clients don’t have regular access to technology. If they don’t have a phone, how are they going to have a computer, or the internet? ... People rely too much on everybody having the same access to everything and that’s not always the case. They need to ask what is the best way to communicate with you? … If there was a way to make all that a little more accessible or more financially affordable for everyone, make some low-cost internet or low-cost computers or something.

The lack of access to technology is a key barrier. Despite the plethora of resources available online, many of these go unused by those who need them most. Jane’s clients were impacted by this lack of access not only from health-related issues but also from being unable to apply for jobs, since many applications need to be completed entirely online.

Lack of financial capital and transportation were also prevalent issues for her clients. Jane expounded that, in the past, clients with Medicaid were able to rely on a taxicab for roundtrip transportation to clinics, but the benefits now only provide transportation one way—forcing clients to choose to get to their appointment or be driven home, leaving many to forego appointments altogether. In addition, related to a lack of technology, only clients who had access to transportation could visit their local libraries for Internet access. Jane would provide transportation for clients and sought out means to provide low-cost phones and other social services to clients in need.

Due to their isolated geographic setting, rural areas present situations in which can people fall through the cracks in accessing services. Carmen, a CHW who works in rural south-central Indiana, explained how she has encountered clients who qualified for health and social services but had been unaware. Carmen described a situation during her volunteer work at a local church helping a client who is an amputee, “I sit down with folks and I’m sitting there going “you’re a veteran”…and I said “you don’t have insulin?”—[he replied] “Well, I know how to make it [insulin] stretch!” I’m like “What!?” I’m going, “you’re not on Medicare?” Carmen further lamented, “how was a caseworker missing this guy? That’s what I’m saying, [CHWs are] reaching them where they are at”. Carmen’s and Jane’s cases illustrate how CHWs are critical in ensuring residents in rural areas are connected with health services.

### 4.2. Immigrant Communities in Indiana

In the southwest region of Indiana, CHWs worked predominantly among Latinx and immigrant communities. Andrés, employed in a small clinic in this region, serves dual roles as a medical interpreter and CHW. Spending half of his day interpreting and the other half doing outreach in the community, he works to promote access, ensure understanding between medical professionals and social service organizations and his clients, and in advocacy. Many Latinx migrant farmworkers pass through the state from April through October harvesting various crops. Andrés had been working with local farmers to develop a mobile clinic, in which he could provide basic health education and social services in addition to partnering with health practitioners to provide basic screenings for diabetes.

Carla, a CHW in the same region, also predominantly provides services for the Latinx immigrant community. Many of her clients are in the process of attaining legal permanent residency or U.S. citizenship. However, even those who have attained permanent residency must wait a minimum of five years before qualifying for federal and state programs including Medicare, Medicaid, and the Children’s Health Insurance Program—highlighting how immigration status, even for those with residency status, is a powerful social determinant of health. As a result, Carla advocates for the needs of these families and seeks to connect them to other resources during this waiting period, describing her advocacy for families in the following way:

I help the parents mostly, really just try to understand the children’s diagnosis or how they need to be treated or what medications they need to be taking. And if people are rude to them, I always stand up for them, too. Because it’s crazy, but a lot of people…they just don’t treat them the same, and I see that on a daily basis.

It is a result of CHWs’ advocacy that their clients are able to learn about and access health and social services. As such, this unique role highlights how advocacy is a form of caregiving. Through advocacy, CHWs in Indiana sought ways to provide health and social services to clients and work with them to identify social determinants of health. CHW advocacy is also present in ongoing discussions of the COVID-19 pandemic in Indiana.

### 4.3. The COVID-19 Pandemic in Indiana

For populations living in rural Indiana, the COVID-19 pandemic has further complicated the present health disparities and structural factors affecting access. The pandemic has closed many resources—such as libraries that provided access to technology—and thereby further isolated already vulnerable rural communities. Moreover, migrant workers—many of them essential workers at various points in the food supply chain—were faced with an increased risk of transmission of the virus.

In April 2020, the Community Health Workers’ Organization of Indiana (CHWOI) began holding public Zoom meetings to discuss issues related to COVID-19. Several CHWs spoke regarding issues of access that their clients had experienced; many explained that the loss of face-to-face time with clients had strained the situation, especially in rural communities, where people lacked Internet access and transportation options. One CHW, who works predominantly with clients suffering from substance abuse disorders in rural, southern Indiana, expressed concern that it seemed as though the ongoing opioid epidemic had disappeared from the public eye and cautioned that once people are unable to access resources (e.g., clean needles), there will be an uptick in behavioral risks and resultant rates of HIV and hepatitis will likely result. Participants in these meetings noted the need for specific action plans to reach rural communities.

Trust, or lack thereof, in the public health system was identified as a major barrier for communities of color. Leticia explained that her clients, who are predominantly African American and Latinx, lack trust in the health system, which further serves as a barrier to care and had led to complications during the pandemic. CHWs also expressed concern about the rollout of the COVID-19 vaccine, noting that clients were already skeptical due to historical precedents such as the Tuskegee Syphilis Study and forced sterilizations in Puerto Rico. Beverly explained that her clients, “are skeptical about it [the vaccine]. We can’t even get them to take the flu shot right now, let alone the COVID-19. The flu shot has been out forever”. As frontline workers, and predominantly women of color, CHWs expressed fears of being “guinea pigs” for the vaccine rollout.

In spite of the limitations associated with the pandemic, CHWs persevered and were inventive in addressing the needs of rural communities. They continued posting on social media for those with internet access, but also sought more traditional methods to distribute information, putting up flyers for resources and information at places community members still frequented during the pandemic, such as the food pantry and grocery stores. Others underscored the need to combat misinformation and help clients and the broader community understand how the virus can still be spread asymptomatically. Another CHW sought to combat a lack of Internet access, working with county supervisors to set up telehealth stations in parking lots that people could drive up to be screened for the COVID-19 virus.

On the organizational end, CHWOI began compiling electronic resources to disseminate to CHWs throughout the state, including basic explanations of COVID-19, populations at risk, how to stem the spread of the pandemic, and how to address clients’ mental health care needs. These meetings have become hubs for CHWs to discuss problems in their communities, how the pandemic has exacerbated or caused new issues to emerge, and how CHWs can—collectively—provide ideas and aid to one another. Additionally, CHWOI has advocated to the state and county health departments to employ CHWs as contact tracers, justified by the extensive knowledge they have of their communities, as well as to facilitate better communication between the state and marginalized communities. This is especially important, as calls and texts from contact tracers are predominantly in English, which may dissuade non-English speakers from answering the phone due to fear, or they may ignore these messages. CHWs can open lines of communication in these communities and stem the spread of the disease among already vulnerable populations [28].

The COVID-19 pandemic has exacerbated pre-existing health disparities in rural Indiana. However, CHWs continued working diligently with clients and communities to assuage these injurious forces, partnering with community leaders, policy makers, and other stakeholders to address these gaps in access and care. In responding to the needs of the clients, fostering relationships, and making positive policy changes in the wake of the pandemic, one CHW, Lucía, asserted “we are at a historic time…we have a great opportunity to do that [cultivate relationships and advocate] and to speak for those who cannot speak for themselves”.

### 4.4. Promotores and Social Determinants of Health in the Rio Grande Valley

Promotores in the Rio Grande Valley of South Texas end up facing many of the same issues as those in Indiana—specifically, negative impacts of social determinants of health, especially high levels of poverty and a landscape that is medically underserved, the confluence of immigration and health policies, and the central role of advocacy as a form of caregiving. However, the historical and political pre-conditions are quite different in the US Southwest—particularly along the US–Mexico border—and the existence of CHW programs here are decades-deep.

In the Rio Grande Valley, the negative impacts of social determinants of health are on full display. The study county (Hidalgo) is one of the most disadvantaged areas of the U.S., with one of the lowest average household incomes in the nation and 33.5% of residents living below the poverty line [53]. Many families reside in unincorporated neighborhoods, called colonias, which often lack roads, water, electricity, and sewage systems. Hidalgo County has the highest number of colonias in the U.S., at an estimated 2300 individual unincorporated and mostly rural neighborhoods. Pollution, crowding, and lack of sanitation in the colonias intersects with gaps in public health services, poor access to care, and high rates of chronic disease make this region among the most medically underserved areas in the nation.

Promotores navigate these colonias like experts and have a long tradition in this region, at least since the 1980s. Their long history of involvement means they have become trusted sources of information and regular partners with local advocacy organizations, frequently implementing health education projects in the region. These promotores are essential in connecting people with low-cost clinics, especially important because colonias are often far-flung from other resources.

Ana is a promotora who supervises an organization that has employed up to 50 promotores at a time (although they currently had 18 hired full-time). She said:

Back in ‘98, we started a pilot program for six months, just to see how it worked, the promotora concept. Ever since then there is no project that we do without a promotora, because it’s that effective and we’re able to take that out to the community. Peer-to-peer is less intrusive and also a little bit more, “I’m willing to listen, because it’s not this person from the outside coming in and pretending that they know better than I do how to raise my children”. It’s their neighbor saying, “Hey look, this is what I learned, let me show you what I learned” kind of thing.

This region also has a long history of segregation and dispossession, a marginalized region in the U.S. where race and ethnicity sharply determine one’s health and access to care. Latinos of primarily Mexican descent account for 91.3% of the population and Spanish is the preferred language [54]. The region has a long history of ethnic separation between Mexican and Anglo residents, with everyday patterns and practices persisting well beyond national legal mandates of desegregation [55]. Through selective and limited incorporation—including but not limited to residential arrangements—racial hierarchies and de facto segregation practices endure.

One of the main strengths of the promotor model is their ability to utilize Spanish language to translate materials and practices and for communicating with clients about health education. Linguistic access can be a major issue in the Rio Grande Valley (where an estimated 83% use Spanish as their preferred language [53]), and often intersects with literacy issues, meaning that simply translating materials into Spanish is not enough. A shared native language further cements the trusted relationship between these promotores and their clients.

Promotores often go door-to-door distributing essential health information and access to residents of colonias. Ana has been a promotora for over 28 years, visiting colonias to connect with women, provide resources on sexual and reproductive health screening, and letting residents know how to apply for funding to receive services free of charge. She says, “It’s a 24/7 job, but I love it”.

### 4.5. Immigrant Communities in the RGV

In south Texas, bridges connecting Texas and Mexico facilitate daily interactions, and communities on both sides of the river have been variously conjoined and split apart through a violent history and changing political identities. Approximately 30% of the population here is foreign-born [53] and an estimated 57% of children—mostly U.S. citizens—live with at least one immigrant parent, markedly higher even than in other counties along the border. In the region’s largest county, some 1 in 10 persons are undocumented [56].

As a result, immigration issues come up frequently in promotores’ interactions in local communities. Heightened border control and lack of public transportation also make this region difficult to traverse, further compounding the obstacles colonia residents face when seeking health care. Promotores frequently make assumptions that their clients or persons in the household have an undocumented legal status. As Luz, a 47-year old CNA and promotora explained,

A lot of times we ask them about their immigration status in other ways or just make it clear we understand their situation. For instance, I might say to someone, “Since you don’t have Medicaid, you can go to [local charity clinic] for this diabetes screening, or whatever”. So, we can ask questions about their insurance coverage or driver’s license or where they work and that can often tell you they are still fixing their papers (*arreglando las papeles*; i.e., they are undocumented).

Promotores are neither surprised nor deterred in their efforts when a client discloses that they have uncertain legal status. In fact, many specifically said that providing some referrals to legal aid is part of their job, referring them to a local advocacy organization that seeks to make immigration services affordable and accessible to the community.

CHWs and promotores in the Rio Grande Valley have a long history of working within other advocacy groups, especially farmworker organizations. Many of the larger promotora programs are affiliated with community-based organizations that have additional foci on labor rights, housing equity, and environmental justice, connecting with specific issues facing colonias. In some cases, these workers simultaneously worked as community organizers. Linda is a 45-year-old single mother of two U.S. citizen sons; she is undocumented but has lived in the United States for more than 30 years. Her primary employment is within a large community-based organization, where she specifically focuses on domestic workers, assisting them with wage theft cases. “I cleaned houses for many years,” she says, “So I know what they are facing. I am passionate about helping them get what is due, in terms of wages. I know what it is like when the employer doesn’t pay”. Five years ago, Linda took a nine-month certification course to become a CHW. Now she is passionate about using these new skills to help women with health issues and has discovered a need for her assistance in cases of intimate partner violence. Again, she could relate to their situation. “They need to talk with someone who has been in that situation. And someone who knows the resources, where to go. So, this has become my life’s work, advocating for them”.

### 4.6. The COVID-19 Pandemic in South Texas

During the COVID-19 pandemic, the deployment of CHWs and promotores was critical as they went into agricultural fields to provide masks, sanitizers, and other protective gear to farmworkers. Promotores also came up with creative strategies to reach out to communities while observing social distancing, such as relying on phone calls rather than door-to-door visits. However, according to one supervisor, Leticia, relying on phone calls makes it very difficult to reach 20,000 people on a regular basis, as they had been used to doing. In addition, as a result of the pandemic, many CHW organizations began to experience financial issues, as patient visits (a metric used by funding agencies) decreased.

Promotores in South Texas were also cross-trained to deliver culturally and linguistically tailored support, resources and information about COVID-19 transmission and safeguards, contact tracing, and also to dispel rampant misinformation. For some colonias without Internet access, information about the pandemic, such as symptoms, testing sites, and social distancing guidelines, can be difficult to access. Promotores step in to bring critical information to disconnected residents, including distributing flyers about drive-thru testing sites.

Promotores also advocated for clients in other tangible ways. When lockdowns associated with the pandemic affected patients’ appointments at low-costs clinics in the region, promotores stepped in to help alleviate the anxiety that colonias residents felt about travel restrictions. They developed a letter that patients could carry with them when requiring essential services, including medical appointments, so they could provide justification for being on the roads during stay-at-home orders. In addition to relying on phone calls and WhatsApp group chats, they started Facebook groups to connect with people (including one named “Las Super Promotoras” with over 600 members. Ana, who supervises a large organization of CHWs in south Texas, commented that,

I think it’s like anything else. An educator is born with that innate want and desire to teach, such that a doctor is born with that innate need to heal. A promotora would say in Spanish, “*Una promotora nace, no se hace*” [a community health worker is born, not made]….that’s just the skills that they bring with them, and all you do is polish some things and how to do reports, how to work within systems and organizations, but we don’t teach anybody how to be a promotora, they already come with that.

## 5. Discussion

CHWs and promotores fill gaps in the provision of care for rural communities experiencing health disparities as the result of structural vulnerability as well as specific concerns related to accessibility and lack of medical providers [3]. In this section, we compare and contrast experiences between the two sites, focusing on the major themes in the Findings relating to CHWs addressing: (1) social determinants of health specifically in rural settings; (2) immigration issues; and (3) the COVID-19 pandemic.

### 5.1. CHWs, Promotores, and Social Determinants of Health in Rural Areas

We return to the key point in recent scholarship that living in a rural area is its own unique health disparity [18,20], presenting a variety of challenges in the pursuit of well-being. Our findings illustrate that in both settings, poverty, a lack of access to technology (specifically, internet), and a lack of transportation complicated access to care. Despite the geographic distance between Indiana and Texas, CHWs and promotores are making significant contributions to the health landscape of rural populations in both states, and are uniquely poised to mitigate the impacts of the social determinants of health, as a myriad of studies have demonstrated [3,16,30,31,32,35]. Examples from these case studies highlight the particular ways in which these workers are effective at drawing on their local knowledge and ingenuity to assuage these deleterious factors. As these workers spend the majority of their time outside of the halls of hospitals and clinics, their knowledge of community resources is an essential factor of their work, as they are able to connect clients across a wide range of needs to resources and organizations.

Advocacy is a crucial role fulfilled by these workers that affects clients at the individual level and can provoke policy change at the community and societal levels. With individual clients, CHWs and promotores engender empowerment by encouraging their clients to seek out resources, speak up during appointments, and advocate for their needs. We assert that advocacy must be viewed as a form of caregiving—a unique component of the CHW/promotor model that benefits health at individual, community, and societal levels. As noted in the case studies, CHWs and promotores engaged in advocacy that included standing up on behalf of clients, disseminating information within the community, and engaging with stakeholders and policy makers. CHWOI in Indiana has advocated for CHWs and their communities, which has resulted in its collaboration with the state government’s response to the COVID-19 pandemic. CHWOI has advocated for the concerns of CHWs, the communities, and the roles CHWs can play in addressing the pandemic.

However, these workers are also affected by a variety of their own structural constraints, such as restrictions in the professional workforce, condescension from other medical professionals, and a general lack of awareness by the broader public [37]. Lack of investment in CHWs negatively affects their ability to do their job, thereby affecting their potential contributions to the health care landscape [37,57]. While these workers can address significant issues in the pursuit of health and well-being in rural settings, they cannot alone be the solution themselves when overarching systemic change is often needed [58]. Nonetheless, facilitating the acceptance of these workers as equal members of professional health care teams can improve the overall health of rural communities. Recent studies have emphasized the importance of stable funding sources [3,59]. This will require increased collaboration between employers of CHWs in both urban and rural environments, in addition to understanding changing demographics of rural populations to effectively tailor the role of these medical paraprofessionals [3,25]. Additionally, protecting and providing support for their advocacy efforts is vital going forward and presents a significant opportunity to address health care needs and promote health equity [60].

### 5.2. Immigrant Communities

Researchers examining rural health disparities must account for the influx of newer communities that are more racially and ethnically heterogenous [6,61]. Many immigrant populations in these two states are deemed essential workers – such as farmworkers and their families – who have specific concerns related to legal status or eligibility for programs, in addition to being at higher risk during the pandemic [25]. For undocumented populations, structural vulnerability inflicted by immigration policy, anti-immigrant rhetoric, and encounters with law enforcement further contribute health insults in the form of discrimination, worry, stress, and fear. Assessing how racial, ethnic, and immigration status [7] influences disparities in rural communities is essential in understanding structural vulnerability.

As our findings show, CHWs and promotores are trusted members within their communities, and thus can address the unique needs of rural immigrant populations. This includes their involvement with linguistically and culturally appropriate health education messaging and ability to link clients with legal aid, and explain complex eligibility requirements for various programs. Many immigrants fear being labeled as a public charge if they use federal- and state-funded programs, which can serve as a barrier to engaging with the health care system. Navigating confusing legal situations can benefit from the role of CHWs and promotores, who provide health education, resources, and advocacy for immigrants in rural communities in order to improve their health and well-being.

### 5.3. CHWs, Promotores, and the COVID-19 Pandemic

This pandemic has exacerbated the already myriad health disparities for rural communities in Indiana and Texas. In both states, rural communities have been significantly impacted by stay-at-home orders, with many communities experiencing additional burdens due to a lack of transportation, technology, and the Internet. The loss of face-to-face interaction has hurt the ability of CHWs to connect with and advocate for their clients. Nevertheless, these workers sought new ways to reach their clients, including video meetings (for those with internet access), phone calls, and face-to-face meetings adhering to social distancing guidelines. Through engaging with community partners, CHWs disseminated information at locations still frequented during the pandemic.

CHW organizations organized regular online meetings for CHWs to discuss issues regarding the pandemic and resources, and thus found themselves at the policy table through repeated efforts and work with local and state politicians. As a result, they have been working with public health officials to bring the issues of their diverse communities to the forefront during planning on how to address the pandemic and the rollout of the vaccine. Addressing lack of trust within marginalized communities will be essential as the COVID-19 vaccine is released. CHWs and promotores are prime partners for governments to assuage fears. These recommendations are applicable for CHWs and promotores across the United States and can serve as a blueprint for their successful integration—and, in doing so, ensure greater success of these workers and fully realizing their potential.

Contact tracing has become an important issue to help track and stem the spread of COVID-19, and is a job particularly well-suited for CHWs and promotores [62,63]. Drawing on their trusting relationships with clients and the community, they can significantly contribute to ending the pandemic [62,63]. This is an important aspect, as many of the communities served by these workers may be hesitant to engage with outsiders. Moreover, hiring CHWs and promotores (and training new individuals in this job) would contribute to job development—especially in communities hardest hit by the pandemic [36,62,64,65].

## 6. Conclusions

The work of community health workers and promotores is vital to improving health and well-being rural communities. As COVID-19 has laid bare fragile health care systems across the globe [29], rebuilding systems that address health care disparities and incorporate new approaches is essential in promoting health equity among all populations. This article has examined how CHWs and promotores fill gaps in the provision of care to rural communities in the United States, illustrating how they confront health disparities using advocacy as a primary tool. We conclude that advocacy must be understood as a form of caregiving, and its role should be bolstered within existing CHW and promotor models.
